# Reimagining Cardiovascular Health and Prevention for Women: The Unrealized Potential of Behavioral Medicine

**DOI:** 10.1016/j.jacadv.2025.102314

**Published:** 2025-11-05

**Authors:** Allison E. Gaffey, Allison J. Carroll, Casey E. Cavanagh, Caroline Y. Doyle, Biswadeep Dhar, Abbey Collins, Hailey N. Miller, Elena Salmoirago-Blotcher, Alyssa M. Vela

**Affiliations:** a Department of Internal Medicine, Yale School of Medicine, New Haven, Connecticut, USA; b VA Connecticut Healthcare System, West Haven, Connecticut, USA; c Department of Psychiatry and Behavioral Sciences and Center for Dissemination and Implementation Science, Northwestern University Feinberg School of Medicine, Chicago, Illinois, USA; d Department of Psychiatry and Neurobehavioral Sciences, University of Virginia School of Medicine, Charlottesville, Virginia, USA; e Department of Psychiatry and Behavioral Sciences, Rush University School of Medicine, Chicago, Illinois, USA; f Department of Human Ecology, University of Maryland Eastern Shore, Princess Anne, Maryland, USA; g Department of Psychology, North Carolina State University, Raleigh, North Carolina, USA; h Johns Hopkins School of Nursing, Baltimore, Maryland, USA; i Department of Epidemiology, Warren Alpert School of Medicine of Brown University, Providence, Rhode Island, USA; j Women's Medicine Collaborative at The Miriam Hospital, Providence, Rhode Island, USA; k Division of Cardiac Surgery, Department of Surgery, Northwestern University Feinberg School of Medicine, Chicago, Illinois, USA

**Keywords:** behavioral, prevention, psychosocial, risk factors, women’s health

Despite being the leading cause of death among women in the United States and worldwide, cardiovascular disease (CVD) remains under-recognized and undertreated among women^1^—reflecting persistent gaps in prevention, timely diagnosis, and evidence-based care. Over half of CVD risk is attributable to modifiable behavioral and lifestyle factors.^2^^,^^3^ Although this may suggest an opportunity for empowerment and personal responsibility, behavioral changes are deeply influenced by social, environmental, and psychological conditions—factors that often place women at a disadvantage. Thus, to improve women’s cardiovascular health, it is essential to address CVD risk within the context of lifestyle and to prioritize behavioral risk factors.

Improving cardiovascular health in women calls for distinct approaches.^4^ The behavioral, physiological, and social factors that shape cardiovascular risk in women are not only different from that in men but are often more complex—emerging from a dynamic interplay of biology, lived experience, and structural inequities. Efforts to reduce the burden of CVD in women must begin with a focus on modifiable contributors to disease onset and progression. A landmark global study identified 9 key modifiable risk factors that together account for over 90% of the first CVD events in women: cigarette smoking, elevated elevated ApoB/ApoA1 ratio (ratio of atherogenic to protective apolipoproteins) ratio, hypertension, diabetes, abdominal obesity, psychosocial stress, poor diet, physical inactivity, and alcohol use.^5^ Intervening on behavioral risk factors also has a significant impact on CVD risk.^6^ These findings underscore the possibility of preventing CVD through comprehensive lifestyle changes and a menu of evidence-based behavioral interventions.

Unique reproductive-related factors—including early and late menarche, infertility, adverse pregnancy outcomes, and absence of breastfeeding—introduce additional layers of cardiovascular risk for women.^7^ For example, 1 in 5 women will develop polycystic ovary syndrome after puberty, a condition linked to insulin resistance, obesity, and metabolic dysfunction, all of which increase CVD risk. Hypertensive disorders of pregnancy and gestational diabetes are robust predictors of future CVD in women, yet these conditions are often excluded from long-term cardiovascular risk assessments. In addition, the use of hormonal contraceptives has been associated with an increased risk of hypertension, thrombosis, and dyslipidemia in women with pre-existing risk factors or those who smoke, which may contribute to long-term cardiovascular vulnerability. Collectively, these examples highlight why reproductive health histories must be integrated into cardiovascular prevention. Although adverse pregnancy outcomes have not enhanced risk prediction models—likely because they occur in younger women—they remain critical to screen for in routine practice.

Although not included among “traditional” risk factors, poor or disordered sleep quality and inadequate sleep duration also disproportionately affect women, particularly during the postpartum period and menopausal transition.^8^ Sleep is an essential modifiable risk factor, which is often overlooked in traditional cardiovascular risk assessment and is now recognized as a critical component of cardiovascular health.^9^ Sleep concerns are often comorbid with psychological distress—including anxiety and depression. Not only are these co-occurring factors more prevalent in women, they are also comorbid with other CVD risk factors and conditions.

In addition to behavioral and biological risk factors, gendered social roles and expectations play a significant role in shaping cardiovascular health for women. For instance, women disproportionately shoulder caregiving responsibilities for children and aging parents. Related role strain can elevate stress levels while simultaneously diminishing women’s time, energy, and financial capacity to prioritize their own health. These social determinants may compound biological vulnerabilities,^10^ underscoring the need for approaches to cardiovascular prevention and care that take into account the full context of women’s lives.

## The role of behavioral medicine

Multiple structural barriers continue to limit the reach of preventive care,^10^ particularly in cardiology, where support for lifestyle change is often infrequent or even absent. Behavioral medicine—a specialty rooted in the integration of behavioral, emotional, and social science with clinical practice—can fill this void. Behavioral medicine specialists, which broadly includes clinical psychologists, social workers, nurses, and psychiatrists and other physicians, have been trained to address the full biopsychosocial context of disease. They can help identify root causes of unhealthy behaviors—food insecurity, housing instability, structural racism, ethnicity-based discrimination, and inadequate health literacy—as cited by the World Health Organization, and develop targeted, practical solutions in partnership with patients and communities. These clinicians employ culturally informed, patient-centered interventions to promote sustainable lifestyle change. Through evidence-based approaches such as motivational interviewing and cognitive-behavioral therapy, behavioral medicine clinicians can significantly enhance patient engagement and outcomes. In an era where 90% of the CVD risk is attributable to modifiable factors,^5^ the health care system can no longer afford to sideline behavioral medicine. Behavioral medicine is not a luxury—it is a necessity for patient-centered cardiovascular prevention—especially among women.

## Cardiovascular behavioral medicine: Gaps in training and support

Despite its benefits, behavioral medicine is generally underfunded and underutilized in medicine and cardiology. To fully realize its potential, significant investments are needed in behavioral training programs, in the expansion of the behavioral workforce, and in the efforts to integrate behavioral medicine into routine care. Integration efforts should prioritize providing structural resources, ensuring behavioral medicine clinicians and transdisciplinary researchers are financially and logistically supported in addressing the broader social and systemic factors that shape cardiovascular risk in women. For example, implementation science can help to increase the reach, adoption, and scale of effective interventions into real-world settings and create a unique opportunity to leverage evidence-based interventions to improve the lives and health of women at risk for CVD.

At the institutional level, few cardiology departments or heart centers employ behavioral medicine specialists, and formal training in lifestyle and behavioral medicine is typically not included in cardiology fellowship programs. Meanwhile, foundations and industry partners have yet to fully embrace the promise of behavioral medicine and generally favor pharmacologic or technological innovations, despite the benefits of behavioral approaches for patients’ risk management and quality of life. Professional societies such as the American Heart Association, American College of Cardiology, Society of Behavioral Medicine, and American Psychological Association should collaborate to champion behavioral medicine as a core component of women’s cardiovascular care.

Further slowing translation into practice, <10% of the National Institutes of Health budget has historically been allocated to behavioral and social research, a fraction that fails to match the immense contribution of modifiable behaviors to chronic disease burden.^2^ This limited funding outlook impedes the development and implementation of behaviorally informed prevention and treatment strategies for both men and women at risk of CVD.

## Priority areas for advancing behavioral medicine role in the improvement of women’s cardiovascular health

To address these multifaceted challenges and improve cardiovascular outcomes for women, a concerted, multisector effort is needed. We propose 2 interdependent priority domains (Table 1).

**Table 1:** Challenges and Opportunities for Behavioral Medicine to Advance Women’s Cardiovascular Health Across Priority Areas

Priority Area	Key Challenges	Opportunities for Behavioral Medicine
1. Clinical and translational research	• Limited integration of psychosocial and behavioral determinants in cardiovascular disease research. • Under-representation of women and diverse populations. • Insufficient data on life stage-specific risk trajectories.	• Incorporate psychosocial variables (eg, chronic stress, social support, sleep quality, discrimination) into longitudinal and intervention studies. • Design and test sex-specific behavioral interventions that address the intersection with cultural factors (eg, race, ethnicity, socioeconomic status). • Leverage implementation science to evaluate the scalability and sustainability of prevention strategies in diverse, real-world settings. • Incorporate behavioral medicine specialists into research.
2. Integrated and accessible care	• Risk assessments overlook behavioral and psychosocial factors that have sex-specific effects on risk and patients’ quality of life. • Gaps in screening and referral for psychosocial and behavioral risk factors. • Limited, routine integration of behavioral medicine specialists and interventions in most cardiovascular medicine care settings.	• Standardize psychosocial risk screening and referral pathways. • Embed behavioral medicine clinicians throughout women’s health care (eg, primary care, women’s health, cardiology). • Implement multidisciplinary care models that address behavioral, psychological, and social factors alongside traditional risk factors. • Expand community-based, telehealth, and peer-led interventions to enhance access to care for underserved populations.

### Integrated and accessible care

An optimized approach to CVD prevention begins with a comprehensive risk assessment that considers, along with traditional risk factors, factors such as sleep quality, social isolation, trauma, caregiving burden, reproductive history, and financial insecurity, which are rarely captured in standard screening but have powerful effects on cardiovascular health and risk. Multidisciplinary care models that include behavioral medicine professionals—which are currently limited to select academic or specialty care centers—should be expanded into cardiology clinics, primary care, women’s health clinics, and community health settings. Such models provide more coordinated patient-centered care and offer greater opportunities for behavior change education and engagement. Nurse practitioners and physician assistants can help extend these models by broadening access, embedding risk screening into routine visits, and reinforcing preventive strategies, particularly in resource-constrained settings or those where physicians’ time is limited. Integration requires not only workflow redesign but also clinician training, patient education, and payer support. Screening for psychosocial risk should be routinized, and referral systems for behavioral health services must be simultaneously expanded and strengthened. Community-based interventions, telehealth models, and peer-support networks can further enhance reach and effectiveness, particularly among underserved populations.

### Clinical and translational research

Bolstering clinical behavioral research is essential to optimize intervention development and implementation. For example, longitudinal cohort studies such as the Women’s Health Initiative, the Study of Women’s Health Across the Nation, and the Nulliparous Pregnancy Outcomes Study: Monitoring Mothers-to-be study have produced invaluable insights into the mechanisms and progression of cardiovascular risk in women. Now, critical gaps remain regarding the prevention and treatment approaches that are most beneficial for women. Behavioral medicine experts are needed to translate these insights into best practices. For example, they can design and test targeted interventions—such as tailored physical activity programs for midlife women experiencing menopausal symptoms, sleep interventions for women with concurrent depression and hypertension, or culturally adapted dietary counseling for diverse populations—and integrate these into primary care, cardiology, and community health settings to improve engagement, adherence, and outcomes. Among women, more evidence is needed to determine the optimal timing for behavioral prevention strategies—particularly in critical life stages such as adolescence, young adulthood, reproductive years and through pregnancy, and across the menopausal transition. Future research must prioritize the inclusion of under-represented populations and explicitly examine how sex and gender interact with race, ethnicity, and socioeconomic status to shape CVD risk, opportunities for intervention, and treatment effectiveness. Funding agencies should support studies investigating the implementation, scalability, and sustainability of evidence-based behavioral strategies in real-world cardiovascular care settings.

## Conclusions

Addressing the cardiovascular health care needs of women requires novel models of care that reflect their lived realities and account for lifetime risk trajectories. Behavioral medicine offers a path forward—evidence-based, patient-centered, and adaptable across the life course—and thus it is uniquely primed to address these factors, particularly through effective integration with women’s health care. Significant and sustained investment is needed in behavioral medicine training, clinical interventions, and research to positively impact women’s cardiovascular health and lifelong care.

## Funding support and author disclosures

Dr Gaffey was supported by a National Institutes of Health/National Heart, Lung, and Blood Institute grant (K23HL168233). The authors have reported that they have no relationships relevant to the contents of this paper to disclose.
